# Nanoindentation Test of Ion-Irradiated Materials: Issues, Modeling and Challenges

**DOI:** 10.3390/ma17133286

**Published:** 2024-07-03

**Authors:** Hailiang Ma, Ping Fan, Qiuyu Qian, Qiaoli Zhang, Ke Li, Shengyun Zhu, Daqing Yuan

**Affiliations:** China Institute of Atomic Energy, Beijing 102413, China; fanping@ciae.ac.cn (P.F.); qyqian1998@163.com (Q.Q.); zql@ciae.ac.cn (Q.Z.); like@tpu.ru (K.L.); zhusy@ciae.ac.cn (S.Z.)

**Keywords:** nanoindentation, ion irradiation, irradiation hardening, zero-point correction, pile-up effect, indentation size effect, crystal plasticity finite element method, multiscale simulation, high temperature nanoindentation

## Abstract

Exposure of metals to neutron irradiation results in an increase in the yield strength and a significant loss of ductility. Irradiation hardening is also closely related to the fracture toughness temperature shift or the ductile-to-brittle transition temperature (DBTT) shift in alloys with a body-centered cubic (bcc) crystal structure. Ion irradiation is an indispensable tool in the study of the radiation effects of materials for nuclear energy systems. Due to the shallow damage depth in ion-irradiated materials, the nanoindentation test is the most commonly used method for characterizing the changes in mechanical properties after ion irradiation. Issues that affect the analysis of irradiation hardening may arise due to changes in the surface morphology and mechanical properties, as well as the inherent complexities in nanoscale indentation. These issues, including changes in surface roughness, carbon contamination, the pile-up effect, and the indentation size effect, with corresponding measures, were reviewed. Modeling using the crystal plasticity finite element method of the nanoindentation of ion-irradiated materials was also reviewed. The challenges in extending the nanoindentation test to high temperatures and to multiscale simulation were addressed.

## 1. Introduction

Nuclear energy is an important contributor to the global ambition of net zero carbon emissions. Advanced structural materials play a critical role in the development of the new generation of cleaner, more efficient, and cost-competitive reactors [1,2,3]. Energetic particles produced during the operation of reactors result in the formation of a series of irradiation-induced microstructural features, such as defect clusters, dislocation loops, cavities, solute segregation, and precipitation, which seriously affect the dimensional and mechanical properties of materials. Irradiation damage may be coupled with other degradation factors, and it can become the most life-limiting factor of various structural materials in fission and fusion systems, as well as materials in nuclear waste disposal systems [1,3,4,5,6,7,8].

The designs of new reactor concepts lead to higher irradiation doses than that of conventional fission reactors [3,6]. Engineering designs must consider the materials used in the anticipated application, so the characterization and evaluation of mechanical properties upon irradiation is one of the main concerns regarding the application of structural materials in nuclear energy systems. It is obviously feasible that the radiation effects can be studied via in-pile irradiation and post-irradiation examination (PIE). However, typical in-pile irradiation lasts from several months to several years, due to the low dose rate. Post-irradiation examination is also time-consuming and challenging, as a result of the high radioactivity of samples after irradiation [9,10,11]. Ion irradiation, which has characteristic advantages such as a high dose rate, little or no residual radioactivity, and precise control over most irradiation conditions (e.g., dose, dose rate, temperature, etc.), offers an indispensable opportunity both in terms of irradiation time, cost, and studying the effects of irradiation parameters. Decades of research have shown that, under appropriate conditions, ion irradiation can reproduce essentially all the microstructural features observed in neutron-irradiated materials [12]. For these reasons, ion irradiations are actively used to simulate the neutron irradiation damages in reactors [13,14].

Among various ions, heavy ions are very efficient at producing dense cascades similar to those produced by neutrons [10]. However, monoenergetic ion irradiation usually means the irradiation damage layer does not exceed several microns. Conventional mechanical testing methods find it difficult to accurately characterize radiation effects on the mechanical properties caused by ion irradiation, especially heavy-ion irradiation, due to a limited damage depth. Therefore, mechanical testing methods at micro/nanoscale have been extensively used to examine the mechanical properties of ion-irradiated samples [15,16,17,18,19]. These methods include micro- and nano-indentation, micro-pillar compression, micro-cantilever bending, and micro-tensile tests. Among them, depth-sensing indentation, i.e., nanoindentation, is the most popular method, because it is easy to manipulate and requires no additional effort in the design and fabrication of micro/nanoscale structures. Exposure of metals to irradiation results in an increase in the yield strength and significant loss of ductility in most cases [20]. Irradiation hardening is also closely related to the fracture toughness temperature shift or ductile to brittle transition temperature (DBTT) shift in alloys with a body-centered cubic (BCC) crystal structure [21]. There are plenty of applications of nanoindentation in ion-irradiated materials [15,22,23,24,25]. It has been suggested that ion irradiation combined with nanoindentation could be a screening method for irradiation hardening [26].

**Figure 1:**
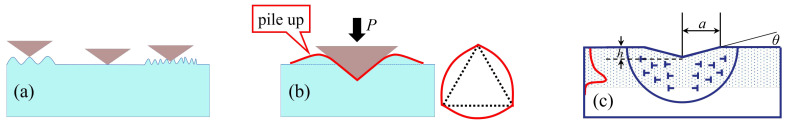
Issues in the nanoindentation testing of ion-irradiated materials discussed in this review. From left to right, (**a**) ill-defined zero-point due to surface roughness and contamination, (**b**) pile-up effect, (**c**) indentation size effect. The expanded plastic zone is depicted as a hemisphere of which the radius is larger than the contact radius *a*. The dotted area indicates the irradiated layer and the red line indicates a typical profile of ion-irradiation dose.

Accurately measured hardness is critical in quantifying the hardening effect of irradiation. Although nanoindentation has evolved to be a standard tool for the measurement of hardness and modulus [27,28], significant uncertainties may arise in the analysis of hardening effects and other mechanical properties using depth-sensing indentation, due to changes in surface morphology and mechanical properties during the irradiation, as well as the inherent complexities in nanoscale indentation, as sketched in Figure 1. Increased roughness [29,30] and contamination [31,32,33] of the sample surface can lead to an ill-defined zero point. Irradiation hardening can cause a change in the mechanical response of materials under the indenter, resulting in different pile-up behaviors before and after the irradiation [34,35,36]. The measured elastic modulus can decrease with indentation depth because of poor mounting of samples [26]. This can be worse for smaller-sized samples in the screening of materials using irradiation hardening as the criteria. Depth-dependent hardening resulting from a non-flat damage profile in ion irradiations, the implanted-ion effect, and the indent size effect in nanoindentations can further complicate the analysis. This has become more challenging as nanoindentation has been extended to high temperatures for ion-irradiated specimens in recent years [37,38]. We give an overview of the abovementioned issues and challenges with the nanoindentation testing of ion-irradiated materials.

This review is organized as follows: In Section 2, after an introduction to the basics of nanoindentation, issues and solutions in the nanoindentation testing of irradiated samples are reviewed. In Section 3, the simulation of nanoindentation for ion-irradiated materials using the crystal plasticity finite element method (CPFEM) is reviewed, with an emphasis on physics-based models. Then, in Section 4, the challenges of extending nanoindentation to high temperatures and multiscale modeling for ion-irradiated samples are discussed.

## 2. Issues and Solutions in the Nanoindentation Testing of Irradiated Samples

### 2.1. Nanoindentation

The standard procedure to perform a nanoindentation has been well established and can found in reviews papers or the ISO/ASTM standards [27,28,39,40,41,42,43,44]. As a reminder, the basic formulae is outlined in the following.

For a sharp indenter of a conical or pyramid shape, the hardness is defined as the maximum load divided by the projected contact area,

(1) $$H=\frac{F_{\max}}{A_{p}}$$

where $F_{\max}$ is the maximum load and $A_{p}$ is the projected contact area. The $A_{p}$ values as a function of contact depth $h_{c}$ are determined by the curve fitting to the modulus of a standard specimen, usually the fused silica [41,45]. For an ideal Berkovich indenter, equivalent to a conical indenter with the half-angle of 70.3° in the projected contact area, the zero-order is $A_{p}=24.56h_{c}^{2}$. In the Oliver–Pharr method, the contact depth $h_{c}$ is expressed as

(2) $$h_{c}=h_{\max}-\varepsilon \frac{F_{\max}}{S}$$

where $h_{\max}$ is the maximum penetration depth, *S* the contact stiffness, and ε is a geometric constant.

In the standard Oliver–Pharr method, the unloading curve is fitted with a power law equation,

(3) $$F=F_{\max}{\left(\frac{h-h_{p}}{h_{\max}-h_{p}}\right)}^{m}$$

where *m*, $h_{\max}$, $h_{p}$ are the fitting parameters. The contact stiffness is defined as the slope at the beginning of the unloading curve,

(4) $$S={\left(\frac{dP}{dh}\right)}_{h=h_{\max}}=\frac{mP}{h_{\max}-h_{p}}$$

The contact stiffness can also be measured using the continuous stiffness measurement (CSM) technique [39]. The geometric constant ε in Equation (2) can be estimated using the power law exponent *m* if the standard Oliver–Pharr method is used. For an indenter with the shape of a paraboloid of revolution, ε=0.75 [41]. An ideal Berkovich indenter has the same contact area with the conical indenter with the half angle of 70.3°, so the ε value is usually very close to 0.75.

The Young’s modulus *E* is calculated by

(5) $$\frac{1}{E_{r}}=\frac{1-\nu^{2}}{E}+\frac{1-\nu_{i}^{2}}{E_{i}}$$

where ν and $\nu_{i}$ are the Poisson ratios of the test sample and indenter tip, respectively. $E_{i}$ is the Young’s modulus of the indenter. For a diamond tip, $\nu_{i}=0.07$ and $E_{i}=1141$ GPa. $E_{r}$ is the reduced modulus of the sample, which is given by

(6) $$E_{r}=\frac{\sqrt{\pi }}{2\beta }\frac{S}{{\sqrt{A}}_{p}}$$

where β is the correction factor that depends on the geometry of the tip [41,46]. For a triangular tip, β=1.034.

### 2.2. Practical Considerations for Nanoindentation Testing of Ion-Irradiated Materials

In practical indentations, the sample is usually mounted on a sample holder with some kind of glue, either thermosetting or fast-setting [47]. Poor sample mounting may cause the deformation of the substrate and lead to extra compliance in the indentation data, so that the measured elastic modulus will decrease with the indentation depth even though there is no gradient in the mechanical properties [34]. The linear relationship between the stiffness and depth for a bulk material can also be used an indicator of the substrate deformation. The substrate deformation can be more severe in the nanoindentation testing of small samples, for example in the mass screening of irradiation hardening. Precautions of firm fixation of the sample or using a clamping system are highly recommended to minimize the substrate deformation.

The thermal drift is generally very small in indentation testing at room temperature. Typical values for thermal drift are <0.05 nm/s in commercial indention testers [47]. If the drift rate is significant, the drift rate can be corrected by adding a segment of constant holding at a suitable place in the force removal curve, as recommended in the ISO-14577 standard [27]. Some testers use an active surface referencing system to reduce the thermal drift, the principle of which basically consists of a reference touching the surface [48,49]. For very small samples or indentation tests near the edge of a sample, an uneven distribution of force on the reference ring can cause incorrect measurement of displacement. Using a sample holder for adjustable height is highly recommended, to ensure that the indenter and the reference sit at approximately the same height.

**Figure 2:**
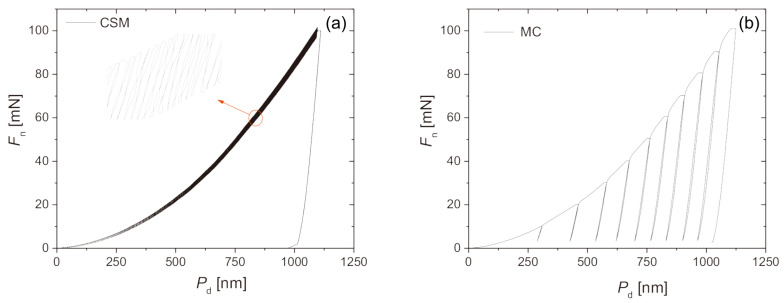
Examples of nanoindentation load–displacement curves measured by CSM (**a**) and MC (**b**). (Reprinted with permission from Ref. [23]).

**Figure 3:**
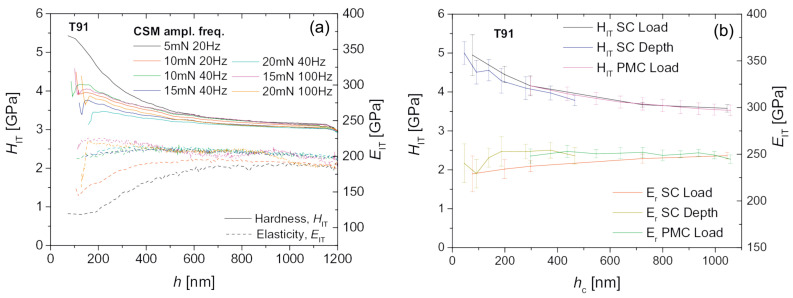
Indentation hardness, HIT, and indentation modulus, EIT of T91 (**a**) measured dynamically by CSM method with different oscillation amplitudes and frequencies. Each curve represents the mean curve of 10 repeats (three to seven repeats for 10 Hz). (**b**) measured quasi-statically with a single cycle (SC) in load control, in depth control, and progressive multi-cycle (PMC) in load control. Each data point represents the average of 15 repeats, with the standard deviation indicated by the error bars. (Reprinted with permission from [23]).

Irradiation damage within the ion-irradiated layer is usually non-uniform. The continuous stiffness measurement (CSM) is the most frequently used method to obtain the depth-dependent hardness and elastic modulus [23,39]. By superimposing a small oscillation on the primary loading signal and analyzing the response of the system, the CSM option enables a continuous measurement of stiffness during loading. A typical load–displacement curve for continuous stiffness measurement is shown in Figure 2a. With a continuous measure of stiffness, the hardness and elastic modulus as a continuous function of depth can be obtained using Equations (2) and (5) from a single indentation. However, the derived mechanical properties are usually dependent on the magnitude and frequency of the oscillation [43], as demonstrated in Figure 3a taken from Ref. [23]. Predetermination of these parameters was suggested, by comparing the results with those in conventional nanoindentation. Quasi-static indentation with multi-cycles (MC) is another method used to obtain the depth-dependent hardness and elastic modulus [23,50], but at the cost of a longer test time. In each cycle, the hardness and modulus can be obtained using the standard Oliver–Pharr method. A typical load–displacement curve for the MC measurement is shown in Figure 2b. It was demonstrated that the multi-cycle indentations gave a good agreement with single-cycle indentations [23], as shown in Figure 3b. If force control is used, a linear or quadratic increment in maximum load for each cycle is usually performed in the loading scheme [23,50]. A quadratic increase in the maximum load as a function of the cycle index would produce results at approximately equal depths [50].

### 2.3. Setting the Zero Point

The zero point is literally defined as the first contact of the indenter with the test-piece surface. The uncertainty in indentation measurement is determined by a number of factors, with the determination of the zero point of contact being first on the list, as stated in the standard ISO 14577 [27]. Two methods were provided in the ISO standard, the first of which defines the zero point as the first increase in either the test load or contact stiffness, and the second of which employs an exponential law to fit the data, up to a maximum of 10% of the maximum depth of indentation. Once the limit has been reached, the indentation depth is set to zero. Commercial nanoindentation test systems, such as the Anton Paar NHT^3^, KLA G200, usually use the first method by default, with other options available [47,48].

The zero point is considerably influenced by the material rigidity, surface forces or contamination, the choice of the parameter threshold, and the sample roughness [51]. For an ion-irradiated sample, carbon contamination [31] can cause headaches in small-scale mechanical testing, not only because of the increased roughness, but also because the resulting zero point determined by the raw indentation data is not the actual first contact with the surface of a pristine sample. Complete suppression of carbon contamination is very difficult, especially for high-temperature irradiation, as the contamination becomes more severe with increasing temperature [32]. It was demonstrated in Ref. [33] that carbon contamination can be effectively eliminated by plasma cleaning of the sample and sample stage, and with incorporation of a liquid nitrogen cold trap, individually and especially in combination. Coating the sample with a thin metallic film may be a less convenient option [33] and can complicate the indentation test. Nevertheless, a cold trap is not always available, and plasma cleaning before irradiation cannot fully prevent carbon contamination.

In Ref. [30], Marteau proposed a method to compensate for the errors caused by inaccurate zero-points. It was shown that the scattering between experimental indentation curves can be minimized using a mathematical optimization that considers all the experimental curves as a whole. However, this method requires a group of indentation data and cannot be applied to an individual indentation.

In the evaluation of the hardening effect by ion irradiation, depth-dependent measurements are usually implemented using either the continuous stiffness test or continuous multi-cycle test. Not only hardness and modulus, but also the contact stiffness at different depths, can be obtained. The zero point can be corrected based on the stiffness–depth curve of a sample with a constant modulus, which is usually the case in ion-irradiated materials. The elastic modulus of a metal with a homogeneous element distribution, e.g., steels, is a reflection of the bonding strength between atoms, which is little affected by microstructure or heat treatment. According to the relationship between the reduced modulus and contact stiffness in sharp-tip indentation, the stiffness can be written as

(7) $$S=\frac{2\beta \sqrt{A(h_{c})}}{\sqrt{\pi }}E_{r}\approx \frac{2\beta \sqrt{C_{1}}}{\sqrt{\pi }}h_{c}E_{r}$$

where the zero-order approximation of the contact area is used. The above equation shows that the stiffness is approximately proportional to the contact depth if $E_{r}$ is independent of $h_{c}$. The zero point can be adjusted by linear fitting the curve of *S* vs. *h_c_*.

Sometimes, $h_{c}$ is not directly provided in some data processing software. Adjustment using the *S* vs. *h_max_* curve is another option. According to the definition of hardness, the contact depth can be expressed as

(8) $$h_{c}=h_{\max}-\varepsilon \frac{F_{\max}}{S}=h_{\max}-\varepsilon \sqrt{\pi }\frac{H\left(h_{c}\right)\sqrt{A(h_{c})}}{2\beta E_{r}}\approx h_{\max}-\varepsilon \sqrt{\pi }\frac{\sqrt{C_{1}}H\left(h_{c}\right)h_{c}}{2\beta E_{r}}$$

In leading order approximation, $h_{c}\propto h_{\max}$, therefore, the zero point can also be adjusted by linear fitting the curve of *S* vs. *h_max_*.

Blunting of the tip also results in an underestimation of contact area [52]. By carrying out zero-point correction based on a *S* vs. *h* curve, the effect of tip blunting on the contact area can be mitigated.

### 2.4. Correction of Pile-Up

Pile-up is the buildup of material around the edge of an indent, as is observed in the indentation of many elastic–plastic materials [28]. On the contrary, a depression around the edge of an indent, i.e., the sink-in effect, is usually observed in brittle materials. In a depth-sensing indentation, the contact area is determined by the analytical relationship with the indentation depth. The contribution of pile-up is not included in the Oliver–Pharr method, which ultimately leads to the overestimation of elastic modulus and hardness.

Indentation pile-up has been extensively investigated in the literature [53,54,55,56,57,58]. Pile-up or sink-in behavior is largely dependent on the strain hardening exponent *n* and the ratio of yield strength to Young’s modulus [56,57,58]. Pile-up is expected for elastic–plastic materials with a small work-hardening exponent, e.g., n<0.1 [57]. Significant changes in mechanical properties can occur during ion irradiation, resulting in different pile-up behaviors before and after ion-irradiation [34,35,59,60]. The correction of pile-up can be critical in the case of a small dose, since the reduced irradiation hardening can be overshadowed by the pile-up, as in the example shown in Ref. [26].

The exact relation between the pile-up and the mechanical parameters is usually difficult to obtain analytically. And the mechanical properties, such as the strain hardening exponent *n* and the yield strength, are not known in advance. The most commonly used methods for determining the actual contact area include post-test measurements using an optical microscope [61], scanning electron microscopy (SEM) [34,59,62], and atomic force microscopy (AFM) [34,52,63,64]. The pile-up correction factor is defined as the ratio of the actual projected area of contact, $A_{p,a}$, to the area calculated from the instrumental contact depth.

Instead of actual observation of the residual indentation impression, Choi proposed a pile-up correction method based on the slopes of loading and unloading curves [52]. However, the elastic work and the power law exponent in the fitting of unloading curve are not always available, for example in continuous stiffness measurements. Based on Equation (6), Heintze et al. proposed an elastic-modulus-based correction (EMC) method [26,35]. The pile-up correction factor can be calculated by

(9) $$C_{\mathrm{EMC}}={\left(\frac{E_{r,c}}{E_{r,a}}\right)}^{2}=\frac{A_{p,a}\left(h_{c}\right)}{A_{p,c}\left(h_{c}\right)}$$

where $E_{r,c}$ and $E_{r,a}$ are the reduced elastic moduli from the instrumental indentation and the actual values, respectively. $A_{p,a}$ and $A_{p,c}$ are the contact areas obtained according to the Oliver–Pharr method and the actual value. In other words, the actual contact depth is corrected by

(10) $$h_{c,a}=h_{c}\sqrt{C_{\mathrm{EMC}}}.$$

The corrected indentation hardness is given by

(11) $$H_{\mathrm{IT},a}=\frac{F_{\max}}{A_{p,a}\left(h_{c}\right)}=\frac{F_{\max}}{A_{p,c}\left(h_{c}\right)}\frac{A_{p,c}\left(h_{c}\right)}{A_{p,a}\left(h_{c}\right)}=\frac{H_{\mathrm{IT},c}}{C_{\mathrm{EMC}}}$$

This method is particularly useful if the elastic modulus can be measured or is known beforehand. In particular, in some cases, an irradiation-independent elastic modulus can be assumed. Heintze et al. used the surface acoustic wave technique to measure the elastic modulus of T91 steel ion-irradiated at 200 °C and W-1 wt% Re alloy ion-irradiated at 300 °C [26,35]. It was found that the elastic modulus was the same for both irradiated and unirradiated material within the error bars of the measurement. The elastic modulus of a material can be strongly affected by cavity defects. It does not change as long as the defects are of the dislocation type [65,66].

### 2.5. Decoupling the Indentation Size Effect from Nanoindentation Hardness

In the micro- and nanoscale indentation tests, the hardness generally shows an indentation size effect (ISE), i.e., the hardness decreases with increasing indentation depth [67,68]. The thickness of the damage layer for an ion-irradiated sample usually does not exceed several microns. The indentation size effect is commonly observed in the nanoindentation of an ion-irradiated material, and has already been discussed extensively, for examples see the review papers [16,17,18,69]. Notably, in the nanoindentation of an ion-irradiated material, there is a damage gradient effect (DGE) and a soft substrate effect. The DGE, in which the bulk-equivalent hardness is dependent on the distance from the sample surface, is due to the depth-dependency of irradiation-induced defects. If the indentation plastic zone extends deep into the unirradiated layer, the hardness will decrease significantly with the indentation depth beyond a threshold depth [24,69,70,71]. These complexities make it very difficult to clean-cut the ISE from the bulk-equivalent hardness.

In bulk materials, the Nix-Gao model, based on the concept of geometrically necessary dislocations (GNDs), has been most widely used to explain the ISE [72,73]. During indentation, GNDs will be generated in the plastic zones under the action of the indenter. The ISE is induced by the strain gradient-induced changes in the density of GNDs.

In the Nix-Gao model, the depth dependent hardness can be described by the following equation:

(12) $$H\left(h_{c}\right)=H_{0}\sqrt{1+\frac{h^{*}}{h_{c}}}$$

where $H_{0}$ is the the hardness in the limit of infinite depth, and $h^{*}$ is the characteristic length. The $H_{0}$ value can be obtained by linear fitting the *H*^2^ vs. 1/*h_c_* curve for nanoindentation data of a bulk material. $H_{0}$ is related to the statistical stored dislocation density

(13) $$H_{0}=3\sqrt{3}\alpha Gb\sqrt{\rho_{s}}$$

where $\rho_{s}$ is the density of statistically stored dislocations (SSDs), *b* the Burgers vector, α the Taylor’s constant, and *G* is the shear modulus, which can be calculated from the elastic modulus and Poisson’s ratio as G=E/2(1+ν). Based on the idea of geometrically necessary dislocations under the indenter, the Nix-Gao model gives $h^{*}$ as

(14) $$h^{*}=\frac{3\bar{r}\tan^{2}\theta }{2b\rho_{s}}=\frac{81\bar{r}b{\left(\alpha G\tan\theta \right)}^{2}}{2H_{0}^{2}}$$

where $\bar{r}$ is Nye’s factor, and θ is the angle between the indenter and the undeformed surface-angle of a conical indenter. The elastic modulus usually does not change when there is no cavity swelling in metals [34,35]. From Equations (13) and (14), in the original Nix-Gao model, the increase in dislocation density after irradiation leads not only to hardening of the material, but also to a decrease in $h^{*}$.

In the original Nix-Gao model, the radius of plastic zone equals the radius of contact. It was later experimentally observed that the storage volume of GNDs is much larger than that given by the contact radius [74,75]. A correction of plastic zone size is necessary to give the correct ISE formulation. In Refs. [74,76], a scaling factor of the plastic zone size with respect to the contact radius was proposed. The improved Nix–Gao relationship can be written as

(15) $$H=H_{0}\sqrt{1+\frac{81\bar{r}b{\left(\alpha G\tan\theta \right)}^{2}}{2f^{3}H_{0}^{2}h_{c}}}$$

where *f* is the scaling factor. The standard Nix–Gao relationship is given by putting f=1. If *s* is the ratio of the plastic-zone radius to indentation depth, i.e., $R_{\mathrm{pz}}=sh_{c}$, then s=f/tanθ. The improved Nix–Gao relationship now reads

(16) $$H=H_{0}\sqrt{1+\frac{81\bar{r}b\alpha^{2}}{2s^{3}h_{c}\tan\theta }{\left(\frac{G}{H_{0}}\right)}^{2}}$$

The typical value of the plastic zone radius was suggested to be in the range of 5∼10 times the indentation depth [17,77,78]. The extent of the plastic zone is dependent on material properties, such as the ratio of hardness to elastic modulus, the work hardening exponent [79,80], as well as tip geometry and indentation depth [34,74]. Direct evidence that the plastic zone size depends on the indentation depth was provided by the transmission electron microscopy (TEM) observation of dislocation networks after indentation [34]. Various formulas have been proposed to give an analytical expression of the plastic zone size [74,78,79,80,81] as a function of material properties and indentation depth. They generally show a decreasing tendency as the hardness of a material increases and the indentation depth decreases.

In order to evaluate the actual hardening from ion irradiation, the indentation size effect must be separated from the indentation size effect as cleanly as possible. The simplest approach to separate out the indentation size effect is to use the ratio or difference in the hardness between the irradiated and unirradiated materials [22,25]. Obviously, the indentation size effect cannot be completely separated out due to the difference in the characteristic length between unirradiated and irradiated samples. Another crude method is linear fitting the $H^{2}$ vs. $1/h_{c}$ curve in the selected region, usually at lower depths where the irradiation hardening is prominent [82,83]. This approach works fairly well for a sample of which the hardness of the ion-irradiated layer reaches saturation, i.e., no damage gradient effect [24]. However, the increase in roughness and contamination during irradiation has a strong effect on the hardness at lower depths. It not only results in a significant increase in the distribution width of indentation data, but also a substantial alternation of the indentation size effect, e.g., reverse indentation size effect (RISE) instead of ISE [84]. And it is difficult to judge unambiguously whether the ion-irradiated layer reaches irradiation hardening saturation. Linear fitting in the selective region, or extrapolation of data to the infinite depth, can lead to significant error in the hardness.

There are several ways to prevent such a problem [18]. The first method is to use variable energies in ion irradiation, to achieve a box-like damage profile [15,26,85,86]. Then, the indentation size effect can be removed with higher fidelity using the standard Nix–Gao model. However, a uniform damage profile is not easy to achieve. The ion energy needs to change multiple times and may be constrained by the accelerator. The second method is to perform indentation on the cross-section of an irradiated sample [15,87,88]. However, sample preparation can be an issue when performing cross-section indentation, because of polishing artifacts and rounding around edges where the polished surface meets the irradiation surface [89].

The third method is to establish an indentation model that compromises the gradient hardening and the indentation size effect, as well as the soft substrate effect [65,71,90]. The ratio of plastic-zone radius to indentation depth *s* is set to a constant in these models, while irradiation hardening can be described by an empirical power-law function of irradiation damage in the unit of displacements per atom (dpa) [65,90,91] or indentation depth [71]. However, since the damage dose in an ion irradiation sample is depth-dependent, not only the irradiation hardening but also the ratio of plastic-zone radius to indentation depth will be dependent on the indentation depth.

In Ref. [70], R. Kasada et al. proposed a formula to extract the depth-dependent bulk-equivalent hardness, as follows:

(17) $$H_{0}\left(h\right)=\sqrt{H{\left(h\right)}^{2}-\frac{1}{h}\frac{dH{\left(h\right)}^{2}}{d(1/h)}}$$

On closer examination, the above formula seems problematic in practice. The derivative $\frac{dH{\left(h\right)}^{2}}{d(1/h)}$ will result in a much larger fluctuation of data. Furthermore, because of the soft substrate effect, the slope of $\frac{dH{\left(h\right)}^{2}}{d(1/h)}$ will be significantly larger than the actual indentation characteristic length as the plastic zone size is well beyond the irradiation layer. The derived bulk-equivalent hardness will be less than that of the unirradiated sample, which is obviously not physical.

Recently, Qian and Ma et al. proposed a simple approach to decouple the indentation size effect from the nanoindentation hardness of ion-irradiated samples, based on a modified Nix–Gao model with an extended plastic zone [50]. This model is more self-consistent with the consideration of the indentation characteristic length and the plastic zone size. A dependency on hardness was introduced for these values, so that their values smoothly alter with increasing indentation depths and naturally reduced to the values of the unirradiated material when the indentation depth is well beyond the irradiation zone.

Qian and Ma assumed that *s* in Equation (16) is a simple power-law function of hardness

(18) $$s\left(h\right)=s_{0}{\left(\bar{H}/H_{u}\right)}^{m}$$

where $s_{0}$ is the ratio of plastic-zone radius to indentation depth for an unirradiated sample, $\bar{H}$ is some kind of averaged hardness in the plastic zone for an irradiated sample, and $H_{u}$ is the hardness of the unirradiated sample. Based on the expanding spherical cavity model, m=−1/3 can be given if assuming a constant modulus and $H=k\sigma_{y}$ [81]. It can be seen from Equation (16) that there is some kind of cancellation between *s* and $H_{0}$ for indentation of an irradiation hardened sample. This might be the reason that a direct ratio of hardness between irradiated and unirradiated samples can be used to evaluate irradiation hardening [22]. From Equations (12), (16) and (18), the characteristic length of an irradiated sample can be approximated by

(19) $$h_{\mathrm{irr}}^{*}=h_{u}^{*}\frac{s_{0}^{3}H_{u}^{2}}{s^{3}{\bar{H}}_{\mathrm{irr}}^{2}}=h_{u}^{*}\frac{H_{u}^{2+3m}}{{\bar{H}}_{\mathrm{irr}}^{2+3m}}$$

It can be seen from Equation (18) and (19) that the plastic size zone parameter *s* and the characteristic length will be naturally reduced to the values of the unirradiated material if there is no irradiation hardening. In the framework of the expanding spherical cavity model, $h_{\mathrm{irr}}^{*}=h_{u}^{*}H_{u}/{\bar{H}}_{\mathrm{irr}}$. Therefore, i is smaller in an irradiated sample than the value of an unirradiated material, as is expected for materials with higher defect density [72]. The weaker size effect was previously observed in Refs. [65,90], from the fitting results of ion-irradiated materials using phenomenological models.

The averaged hardness largely free of the indentation size effect can be calculated by solving or iterating the following equation:

(20) $${\bar{H}}_{\mathrm{irr}}=\frac{H_{\mathrm{irr}}}{\sqrt{1+\frac{h_{\mathrm{irr}}^{*}}{h_{c}}}}=\frac{H_{\mathrm{irr}}}{\sqrt{1+\frac{h_{u}^{*}}{h_{c}}\frac{H_{u}^{2+3m}}{{\bar{H}}_{\mathrm{irr}}^{2+3m}}}}.$$

During iteration, the characteristic length of an unirradiated sample is set to be the initial value of $h_{\mathrm{irr}}^{*}$. In the above equation, the hardness obtained at one depth is irrelevant to the hardness at other depths, thus avoiding the unpleasant effects of increased roughness and contamination at lower depths.

**Figure 4:**
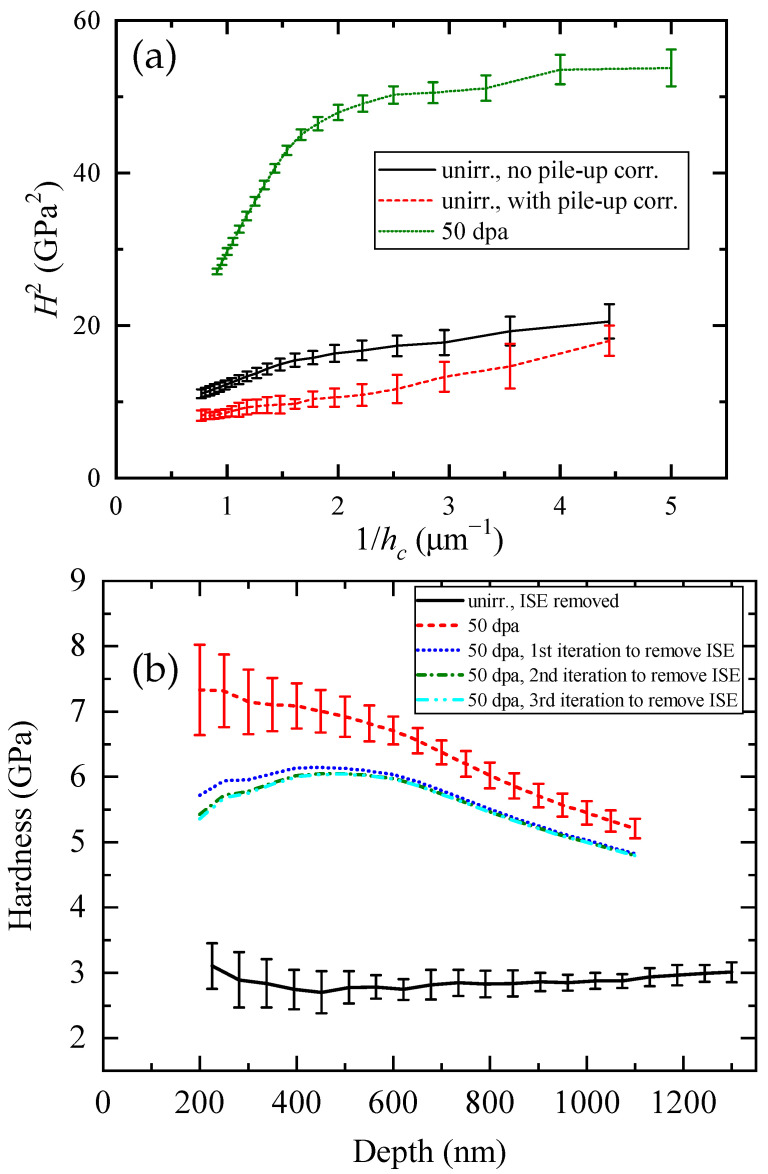
(**a**) $H^{2}$ as a function of contact depth for indentations on the unirradiated and irradiated region of the 9Cr-FMS sample. (**b**) The averaged hardness after the pile-up correction and removal of ISE.

An ion-irradiated ferritic/martensitic steel (FMS) was taken as an example to test this solution. The sample was irradiated to a peak dose of 50 dpa by 40 MeV Fe at 350 °C. After the correction of pile-up, the $H^{2}$ values were drawn as a function of 1/h in Figure 4a. By linear fitting the $H^{2}$ vs. 1/h curve with pile-up correction, the hardness of unirradiated material and characteristic length were determined. Using Equation (20), the hardness values free of ISE were as shown in Figure 4b. The curve of the unirradiated region is flat as a function of depth, as expected. As for the curves of the irradiated region, it was shown that three iterations of Equation (20) already gave a convergent result. A hardening bump is observed in the decoupled nanoindentation hardness of the ion-irradiated 9Cr-FMS. Beyond the bump, the hardness gradually dropped as the soft substrate was taken into effect. From the peaks of dpa and hardness, it was estimated that the plastic zone was about 8.6 times the indentation depth for the irradiated region. The hardness at 200 nm was only about 0.6 GPa lower than the peak hardness. This is a sign of approaching saturation, commonly observed in FM steels. The corresponding damage was 4 dpa at 1700 nm when the estimated ratio of the plastic zone to indentation depth was used. This is also consistent with data from typical in-pile irradiated FMS samples [92].

### 2.6. Models for Extracting Bulk-Equivalent Hardness

In Section 2.5, several methods for decoupling the indentation size effect were reviewed. However, the plastic zone under the indenter can stretch far beyond the indentation depth. The calculated hardness from Equation (17) or (20) actually represents an averaged mechanical property over the whole plastic zone. The ISE coupled with DGE and soft substrate effect further increase the complexity in the extraction of the actual irradiation hardening corresponding to the bulk material.

**Figure 5:**
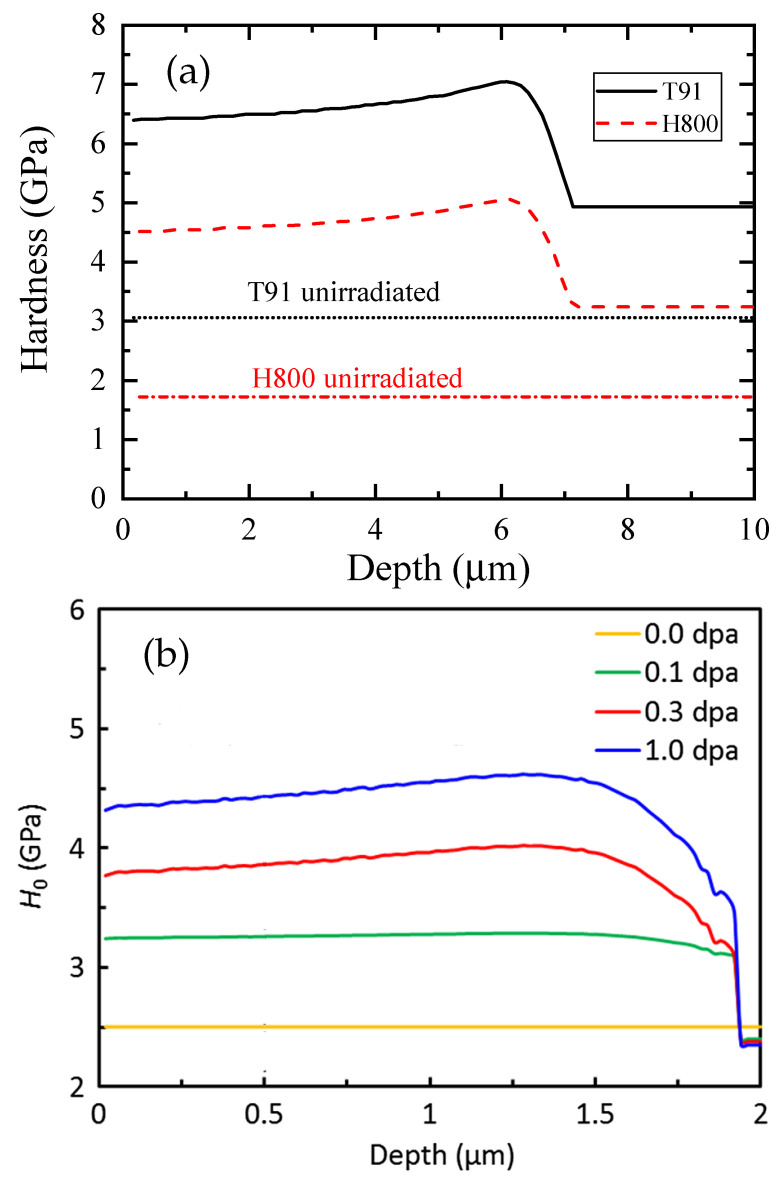
Actual hardness profiles as a function of depth reconstructed from the fitting parameters and dpa profile. (**a**) A ferritic/martensitic steel T91 and a austenitic steel 800H irradiated with 70MeV Fe^2+^ to a peak damage of 60 dpa at 452 °C. Adapted from Ref. [65]. (**b**) A 22NiMoCr3-7 type reactor pressure vessel (RPV) steel irradiated with 5 MeV Fe^2+^ to different doses at 300 °C. Taken from [90] (Reprinted with permission from [65,90]).

Several phenomenological models were developed to extract the actual hardness at different depths of ion-irradiated materials [65,90,93]. Most of these models are based on the volume fraction model and assume a hemispherical shape for the plastic zone, although in reality the shape is much more complicated, as revealed by TEM observations [34,88]. In the volume fraction model, the hardness from an indentation test is taken as the averaged hardness in the plastic zone,

(21) $$\langle H\rangle \left(h_{c}\right)=\frac{3}{2R(h_{c})}\int_{0}^{R(h_{c})}\left\{1-\frac{z^{2}}{R^{2}}\right\}H\left(z\right)dz$$

where $R_{\mathrm{pz}}\left(h_{c}\right)=sh_{c}$ is the radius of the plastic zone at the contact depth $h_{c}$ and H(z) is the hardness at the depth *z*, respectively. To include the indentation size effect in the above equation, H(z) is usually written as [90]

(22) $$H\left(z\right)=\left(H_{u}+\Delta H_{\mathrm{irr}}\right)\sqrt{1+\frac{h^{*}}{h_{c}}}$$

where $H_{u}$ is the hardness of the unirradiated material and $\Delta H_{\mathrm{irr}}$ is the irradiation-induced hardness increase that can be described by the irradiation hardening law and the irradiation dose profile. A. Kareer et al. used a slightly different formula, where $H\left(z\right)=H_{u}\sqrt{1+h^{*}/h_{c}}+\Delta H_{\mathrm{irr}}$. In Refs. [65,90], the power law function of irradiation hardening with respect to the irradiation dose was adopted. This relationship can be easily replaced by Makin’s hardening law, in which hardening saturation is introduced [94]. The *s*, $h^{*}$, $H_{u}$ and the parameters in the irradiation hardening law can be fitted using a non-linear least-squares fitting routine. The actual hardness as a function of depth can be recalculated using the fitting parameters and the dpa profile. Figure 5 shows examples of ion-irradiated T91 and 22NiMoCr3-7 steels from Refs. [65,90].

There are two inconsistencies in the phenomenological models introduced in Refs. [65,90]. $H_{u}$ is set as a parameter to be fitted for the irradiated materials, which could be inconsistent with the value determined from the measured hardness of the unirradiated material, see Figure 5a which was adapted from Ref. [65]. In addition, the authors used an overly simple assumption that the characteristic length and the ratio of plastic zone size to indentation depth are independent of hardness or depth, with another inconsistency being that their values should approach the values of the unirradiated material when the indentation depth is larger than the thickness of the irradiated layer. As discussed in Section 2.5, Qian and Ma introduced a hardness dependency on the size of the plastic zone as well as the characteristic length, so that they are naturally reduced to the values of unirradiated material. This concept can be combined with a volume fraction model to give a phenomenological indentation model with improved self-consistency.

In Refs. [71,91,95,96], Xiao et al. and Vogel et al. replaced the phenomenological hardening law with the dispersed barrier hardening (DBH) model, which can link the irradiation hardening with the density of dislocation loops [97,98]. Irradiation hardening can be written as

(23) $$\Delta H_{\mathrm{irr}}=M\alpha \mu b\sqrt{N_{d}d_{d}}$$

where *M* is the Taylor factor, α is the defect cluster barrier strength, μ is the shear modulus, *b* is the magnitude of Burgers vector, and $N_{d}$ and $d_{d}$ are the defect cluster density and diameter, respectively. The mean size of the dislocation loops was taken from the experimental value. In these studies, the defect density was parameterized using a power law function of depth instead of parameterized according to the dpa profile. Xiao and his collaborators also discussed the square superposition hardening models in Refs. [95,99]. Comparing with the linear superposition model in Equation (23), it was concluded that the square superposition hardening model works better for those data where the indentation depth exceeds the maximum irradiation depth.

In nanoindentation tests, the plastic zone can reach a size of 5∼10 times the indentation depth. As a result, the plastic zone may involve multiple grains at large indentation depths. The grain boundaries may play an important role in the determination of hardness, especially for nanocrystalline materials [100,101]. In order to extract irradiation hardening from ion-irradiated polycrystaline materials, the grain size effect was introduced into Xiao’s mechanistic model in Ref. [95]. In the framework of the modified model, the grain size effect can be separated from the contribution of dislocation hardening.

The above discussions primarily focused on the nanoindentation testing of ion-irradiated bulk materials. Coatings have also been extensively studied as a protective barrier against oxidation and corrosion for the fuel claddings used in light-water nuclear reactors [102,103,104] and the core structural materials in future nuclear energy systems [105,106]. It is more complicated to analyze the indentation hardening for ion-irradiated materials with coatings, especially with multilayer coatings. This is beyond the scope of this review, as we refer to the following works for detailed discussions [107,108,109].

## 3. CPFEM Modeling the Nanoindentation of Ion-Irradiated Materials

The finite element method (FEM) has been the most widely used indentation test to understand the indentation process, and the stress and strain fields under the indenter tip for various materials. For an example see Ref. [110], where the FEM simulation of indentation using a conventional elastic–plastic constitutive model was reviewed. Finite element modeling of nanoindentation for ion-irradiated materials using conventional constitutive models was performed in [111]. However, it is not a trivial task to develop a microscopic-mechanism based FEM model to simulate the indentation of ion-irradiated materials. The traditional constitutive model is based on the classical plasticity theory of continuum. It does not include any scale factor related to material properties, that is, the intrinsic scale parameter of the material, so it cannot simulate the size effect at micro-nano scale. Depth-dependent hardening resulting from the non-flat damage profile in ion irradiation and the complex response of irradiation in materials further complicates the analysis.

The crystal plasticity finite element method (CPFEM) incorporates crystal plasticity constitutive equations (crystallographic slip, lattice rotation, rate-dependent hardening model, etc.) into a finite element framework [112]. The CPFEM has the advantage of bridging the microscopic defects to the macroscopic mechanical response. Irradiation hardening is generally assumed to be proportional to the increase in critical stress due to irradiation-induced defects. To model irradiation hardening at the microscopic level, the interaction of dislocations with the irradiation-induced defects can be introduced into a physics-based crystal plasticity finite element (CPFE) model. The radiation effects in metallic materials using the crystal plasticity theory were reviewed in Ref. [113]. As for the nanoindentation of ion-irradiated materials, various efforts have been devoted in recent years to realizing viable CPFEM modeling [69,85,88,114,115,116,117,118].

In this section, the application of CPFEM to the nanoindentation of ion-irradiated materials will be briefly reviewed, with an emphasis on physics-based constitutive models.

### 3.1. The CPFEM Framework: Phenomenological vs. Physics-Based Constitutive Models

The crystal plasticity theory is established on continuum mechanics. In the finite strain framework, the deformation gradient can be decomposed into elastic and plastic parts, where the plastic part is more of concern in CPFEM. To describe the deformation dynamics, the changing rate of the deformation gradient, i.e., the deformation velocity gradient *L*, was introduced and can be written as

(24) $$L_{p}=\sum_{\alpha =1}^{N}{\dot{\gamma }}^{\alpha }m^{\alpha }⊗n^{\alpha }$$

where *N* is the number of active slip systems. $m^{\alpha }$ and $n^{\alpha }$ are, respectively, the unit vectors describing the slip direction and the slip plane normal of the slip system α. ${\dot{\gamma }}^{\alpha }$ is the shear rate of this system, which is determined by the constitutive model. Therefore, the connection between macroscopic continuum mechanics and crystal plasticity constitutive model can be established.

The constitutive model mainly focuses on the relationship between the applied stress and the shear rate. Existing constitutive models can be categorized into phenomenological constitutive models and physics-based models. In phenomenological constitutive models, the plastic shear rate ${\dot{\gamma }}^{\alpha }$ is determined by the resolved shear stress and critical resolved shear stress. The power law model suggested by Rice [119] and Peirce et al. [120] is the most commonly used formulation,

(25) $${\dot{\gamma }}^{\alpha }={\dot{\gamma }}_{0}{\left|\frac{\tau^{\alpha }}{\tau_{c}^{\alpha }}\right|}^{\frac{1}{m}}\mathrm{sgn}\tau^{\alpha }$$

where $\tau^{\alpha }$ is the resolved shear stress of the slip system α, ${\dot{\gamma }}_{0}$ and *m* are material parameters that determine the reference shear rate and the rate sensitivity of slip, respectively. $\tau_{c}^{\alpha }\left(\mathrm{CRSS}\right)$ is the critical resolved shear stress (CRSS), the criterion for the activation of the slip system. For the phenomenological constitutive model, $\tau_{c}^{\alpha }$ can be expressed as

(26) $${\dot{\tau }}_{\mathrm{CRSS}}^{\alpha }=\sum_{\beta =1}^{n}h_{\alpha \beta }\left|{\dot{\gamma }}^{\beta }\right|$$

where $h_{\alpha \beta }$ is the hardening matrix, the function of which can also be written phenomenologically.

One of the critical drawbacks of the phenomenological models is that the material state is only described in terms of the critical resolved stress and there is no link to microscopic structures, such as defects or dislocations. Based on the conventional crystal plasticity theory, it is possible to introduce GNDs and SSDs into the hardening formula in the CPFEM framework, leading to mechanism-based strain gradient crystal plasticity (MSG-CP) theory [121,122,123,124]. However, MSG-CP is not fully physics-based. Various groups have developed a fully physics-based constitutive model [112,125,126]. Based on the physics-based formalism, the creation and annihilation of dislocations, and the evolution of GNDs and SSDs during the indentation process, can be taken into account [127,128]. Therefore, a physics-based CPFE model that captures the essence of CPFEM is the ultimate solution for simulating the nanoindentation of ion-irradiated materials.

For a physics-based constitutive model, the shear rates ${\dot{\gamma }}^{\alpha }$ is linked to the density $\rho_{m}^{\alpha }$ and the average velocity $v^{\alpha }$ of mobile dislocations. Under the assumption of forest cutting as the rate determining process, $v^{\alpha }$ can be expressed as a function of the effective shear stress $\tau_{c}^{\alpha }$, which can be calculated from the resolved shear stress and the passing stress as

(27) $$\tau_{\mathrm{eff}}^{\alpha }=\left\{\begin{array}{cc} \left|\tau^{\alpha }\right|-\tau_{\mathrm{pass}}^{\alpha }=\left|\tau^{\alpha }\right|-c_{1}Gb\sqrt{\rho_{P}^{\alpha }+\rho_{m}^{\alpha }} & \left|\tau^{\alpha }\right|>\tau_{\mathrm{pass}}^{\alpha }\\ 0 & \left|\tau^{\alpha }\right|\leq \tau_{\mathrm{pass}}^{\alpha } \end{array}\right.$$

where $\tau_{\mathrm{pass}}^{\alpha }$ is the passing stress, which is caused by the parallel dislocations $\rho_{P}^{\alpha }$, and $\rho_{m}^{\alpha }$ is the mobile dislocations. *b* and *G* are the burgers vector and shear modulus, respectively. Therefore, the microscopic dislocation densities $\rho_{P}^{\alpha }$ and $\rho_{m}^{\alpha }$ are incorporated into the CPFEM framework and used to describe CRSS. In the physics-based constitutive mode, the contribution to flow stress from other microstructure-dependent components, such as grain-boundaries, can be added to capture additional hardening effects.

### 3.2. Application of CPFEM in Nanoindentation for Ion-Irradiated Materials

Simulating single crystal nanoindentation through CPFEM has become relatively mature using a phenomenological constitutive model. For example, nanoindentation for face-centered cubic (FCC) copper and aluminum single crystals was simulated using a classic Bassani–Wu hardening model in Ref. [129]. For polycrystals, a representative volume element (RVE) can be used to calibrate the crystal plasticity constitutive parameters, similar to the CPFEM modeling of macroscopic mechanical tests [130]. The influence of grain orientation and grain boundaries on the nanoindentation was studied using the polycrystalline CPFEM. By introducing GNDs into the classical hardening model, the rendered MSG-CP theory [122,123] is able to study the indentation size effect in nano-micro scale indentations [124,131,132].

**Figure 6:**
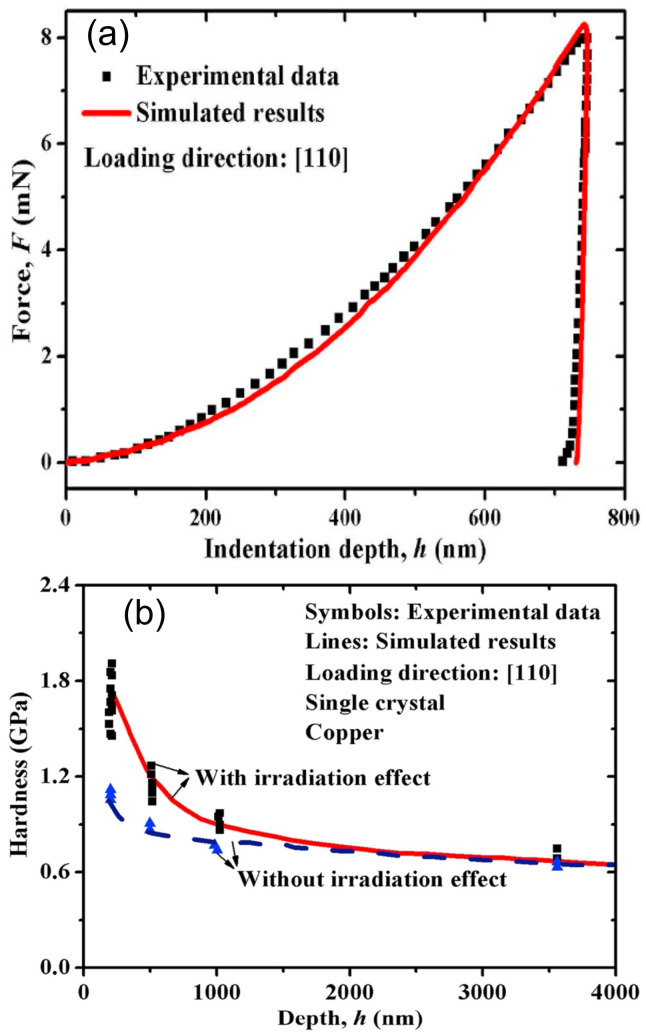
(**a**) Load vs. indentation depth of unirradiated single-crystal copper at the [1 1 0] loading direction. (**b**) Hardness vs. depth for the unirradiated and ion-irradiated single-crystal copper. The sample was irradiated with 200 keV proton at room temperature [18]. The thickness of the ion-irradiated layer was 1 μm. (Reprinted with permission from [116]. Copyright 2019 by Elsevier).

Xiao et al. used the MSG-CP model to simulate the nanoindentation behavior of ion-irradiated single crystal copper [116] and plasma-exposed tungsten [133]. In Ref. [134], the hardening of a single irradiated tungsten crystal was studied with the MSG-CP model. In order to study both the ISE and irradiation hardening simultaneously, GNDs and irradiated-induced hardening were incorporated in the CPFE models. The irradiation hardening due to irradiated-induced defects was taken into the constitutive model using the dispersed hardening model, similarly to the discussions in Section 2.6. The non-uniformly distributed defects generated in the ion-irradiation were linked to the dpa profile, which could be determined through binary collision approximation codes, such as TRIM [135]. The hardness–depth relationships of the unirradiated and ion-irradiated materials could be well reproduced using the MSG-CP model, see Figure 6 for a comparison.

In the framework of MSG-CP theory, other deformation mechanisms such as twinning and creep can be added to the MSG-CPFEM models [136,137,138,139]. In Ref. [140], Wang et al. studied the irradiation hardening effect of pure zirconium metal using ion-irradiation and a MSG-CPFEM model. They achieved a relatively uniform irradiation layer in Zr, with multiple-energy ion irradiation. The deformation mechanisms in the nanoindentation of unirradiated and ion-irradiated Zr were studied using the MSG-CPFEM. The load-displacements of the simulated grains under both non-irradiated and irradiated conditions were successfully repeated using the CPFEM method. The CRSS of each deformation system was obtained based on the simulations. With the help of the MSG-CP model, it was shown that the irradiation hardening shows a strong orientation dependence on Zr.

Irradiation hardening is generally assumed to be proportional to the increase in the critical stress due to irradiation-induced defects. To describe irradiation hardening at the microscopic level, the interaction of dislocations with the irradiation-induced defects must be introduced, which may include defect clusters, dislocation microstructures, irradiation-induced segregation, and precipitation. The physics-based CPFEM provides an excellent framework bridging the microscopic defects and macroscopic mechanical behavior. In Ref. [141], authors developed a physics-based CPFE model to study the nanoindentation process of an ion-irradiated A508-3 steel. Dislocations were divided into mobile dislocations and immobile dislocations. Mobile dislocations can move through slip systems in a thermally activated manner, and their rate of movement is affected by temperature, stress, and internal obstacles in the material (such as irradiation defects). Immobile dislocations also contribute to the hardening effect of the material. Irradiation defects constitute new obstacles in irradiated materials, mainly including dislocation loops, solute clusters, and defect clusters. In the model, the dislocation loop is treated as the main source of hardening and the other defects are simply treated as equivalent to dislocations. By taking into account the evolution of these dislocations, the changes in the mechanical properties of the material after irradiation were well described by the model.

**Figure 7:**
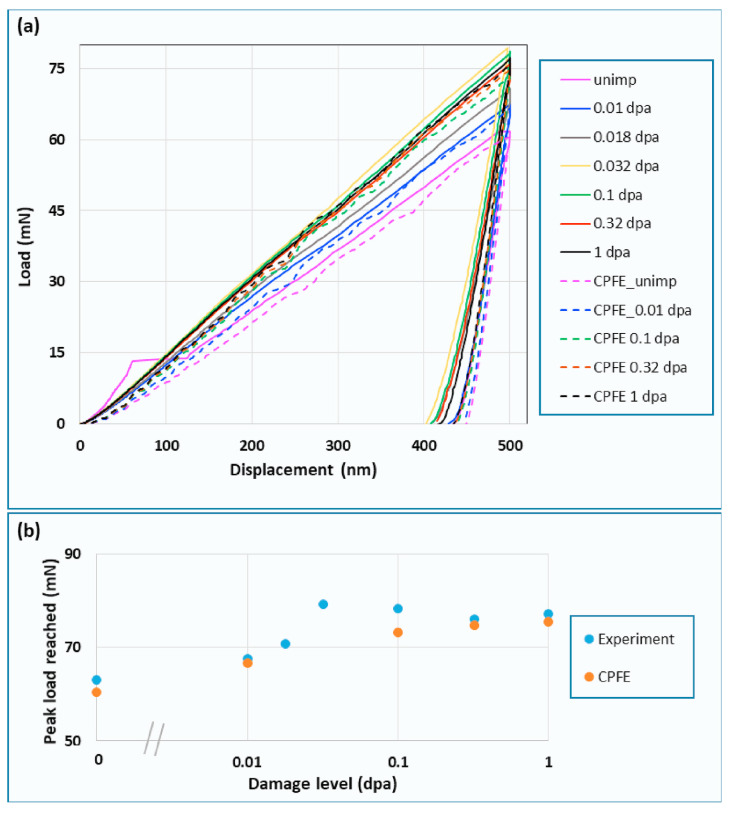
(**a**) Load–displacement curves as measured by nano-indentation and predicted by CPFEM for <0 0 1>-oriented grains in self-ion implanted tungsten. The tungsten sample was irradiated with 20 MeV tungsten to various dpa at room temperature. Nanoindentation of the unirradiated and irradiated samples was carried out with a spherical indenter tip of radius ∼5 μm to a maximum indentation depth of 500 nm. (**b**) Plot of peak load reached by the ion-implanted samples with different damage levels as measured by nano-indentation and predicted by CPFEM. See Ref. [36] for details. (Reprinted with permission from Ref. [36]. Copyright 2020 by Elsevier.)

**Figure 8:**
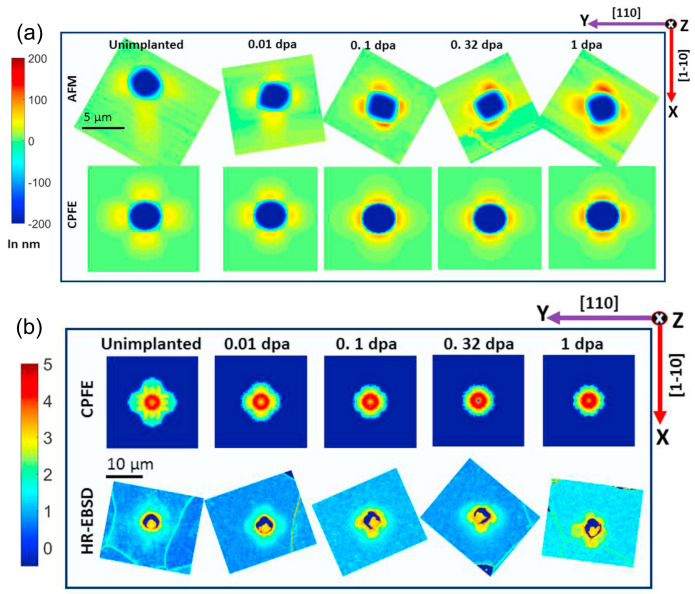
(**a**) Surface morphologies (height profile) of nanoindents in unirradiated and ion-irradiated tungsten as predicted by CPFEM and measured by AFM after indentation. The surface normal pointing out of the page is along [0 0 −1]. The color scale shows the surface height in nm. (**b**) The densities of GNDs over all slip systems as predicted by the CPFEM and measured by the high-angular resolution electron backscatter diffraction (HR-EBSD) technique. The plots are shown on the sample surface i.e., on the XY cross-section. The same color scale is used for all plots showing $\log_{10}\left(\rho \right)$ with ρ in 1/$\mu m^{2}$. See Ref. [36] for details. (Reprinted with permission from Ref. [36]. Copyright 2020 by Elsevier.)

In Ref. [36], the deformation behavior of ion-irradiated tungsten was studied using spherical nanoindentation and physics-based CPFE simulations. Their CPFE model explicitly accounts for the interaction of glide dislocations with irradiation-induced dislocation loops. The CPFE predictions of load–displacement curves, surface morphologies, and GND densities around the indents were compared with experimental data. The authors also studied the orientation dependence of deformation behavior. As can be seen from Figure 7 and Figure 8, it was shown that the physics-based CPFE simulations not only reproduced the load–displacement curves but also correctly captured the dependence of pile-up behavior on the irradiation dose and the grain orientation. It was demonstrated that the CPFE model captured the underlying interaction mechanism between the irradiation defects and glide dislocations. After the verification of the CPFE model, the authors constructed a polycrystalline tungsten sample. The macroscopic deformation behavior of the irradiated bulk tungsten was predicted based on the CPFE simulation.

## 4. Challenges in the Nanoindentation Testing of Ion-Irradiated Materials

### 4.1. Challenges of Extending Nanoindentation to High Temperatures

Although nanoindentation has become a standard tool in the evaluation of hardening in ion-irradiated materials, most nanoindentation tests of ion-irradiated samples were performed at room temperature, while the core structural materials in nuclear reactors work at high temperatures for most of their service life. The mechanical properties of materials are heavily dependent on temperature. In the assessment of radiation effects on mechanical properties, test data under the operating temperature are even more valuable. However, it remains challenging to extend nanoindentation tests to high temperatures [37,38,142,143]. The main challenges include the thermal stability of the instrument, management of thermal gradients and thermal drift, and the chemical stability of the indenter materials and samples. Other issues include the data analysis when the material is severely plastically deformed at high temperatures, and the lack of reference samples used to calibrate the instrument at high temperatures. Addressing these challenges is key to pushing the upper temperature limit of the high-temperature nanoindentation technique. The challenges and solutions of high-temperature nanoindentation were summarized in Ref. [144], as shown in Table 1. In addition to the above challenges, there is one issue that has no immediate solution. There is no available standard reference for high-temperature hardness. Thus, the variation in the geometric function of the tip with temperature has not been considered so far. Nevertheless, it is generally safe to compare the relative change in materials.

Through continuous research for more than two decades, the design of high-temperature nanoindentation testers has improved significantly in recent years, especially in terms of heating and thermal management, indenter materials, and thermal drift suppression and data analysis [37,38,142,145,146,147,148]. In the development of high-temperature nanoindentation devices, in situ high-temperature nanoindentation in a scanning electron microscope (SEM) has become most appealing, as SEMs offer a high vacuum that in the first place prevents the oxidation or degradation of the sample and the tip, and automatically provides the vibration damping necessary for nano-scale measurements. An SEM enables direct positioning and in situ observation of deformation under the tip, as well as monitoring the possible damage of the tip [144]. Commercially available systems can now perform stable indentation testing at temperatures up to 800 °C with thermal drift levels similar to those present at room temperature. Indentation up to 1100 °C has been achieved in the laboratory [148].

With the significant progress in the high-temperature indentation technique, there has been continued interest in high-temperature indentation for nuclear fission/fusion relevant materials. However, works on high-temperature indentation on ion-irradiated materials are rare. In Ref. [149], nanoindentation was performed on helium-implanted tungsten at temperatures up to 750 °C. A softening of above 300 °C was observed, in agreement with the results from the micro-scale high temperature indentation. It was demonstrated that the test temperature had a significant effect on the hardening effect of the helium-implanted tungsten. The hardening was negligible above 450 °C. In Ref. [38], unirradiated and ion-irradiated 800H steels were tested up to 300 °C using a more conventional setup of hot indentation. The effect of temperature on the indentation size effect was studied, and it was found that the indentation size effect was less pronounced at high temperatures due to the increase in the plastic zone size.

### 4.2. Challenges in the Multiscale Simulation of Nanoindentation for Ion-Irradiated Materials

The changes of the mechanical properties in irradiated materials are directly related to the radiation-induced microstructural changes. In the CPFE models established so far, only the radiation defects related to dislocations have been considered. Besides the radiation defects of dislocation type, the mechanical properties are also related to other defect clusters, such as stacking-fault tetrahedrons, cavities, solute segregation, and precipitation. These defects are simply treated as equivalent to dislocations in these CPFE models and have yet to be addressed. In addition, the density of defect clusters in these CPFE models was introduced through experimental observations or by fitting the indentation hardness in the simulation to experimental values. Defect clusters are actually built up with increasing dose during irradiation. Simulation at lower levels is needed to fully understand the evolution of different defect clusters and the changes in mechanical property in ion-irradiated materials.

The physical processes involved in radiation damage, and its effects on mechanical properties, are inherently multiscale and hierarchical [150]. The spans of length and time can scale from an atomic size to continuum and picoseconds to decades. Therefore, detailed simulation of radiation defects should also be fundamentally multiscale, from a quantum-mechanics atomistic description, to classical atomic motion (molecular dynamics, MD), diffusion and transport of defects in the meso and microscopic scales (Kinetic Monte Carlo/KMC, rate theory/RT), interaction of defects and dislocation (dislocation dynamics, DD), and finally the CPFEM. It remains a challenge to connect the different levels of simulation during irradiation processes. Multiple collaborative efforts have contributed to multiscale simulations in recent years, to understand the radiation effects of materials from fuels, structural materials for fission and fusion systems, to nuclear wastes [151,152,153]. The topics in this field have been intensively reviewed, see Refs. [113,150,151,154,155,156,157,158], although it remains far from mature.

In the description of the long-term evolution of defect clusters, rate theory (RT) [159,160] and object kinetic Monte Carlo (OKMC) methods [161,162] are the most popular choices. It is worth noting that there have already been attempts to integrate the rate theory with crystal plasticity models, to simulate the mechanical behavior of neutron-irradiated materials, both for quasi-static tensile behavior [137,163] and time-dependent behavior such as creep [137,139,164]. There have also been attempts to combine lower scale simulation with CPFE models. In Ref. [134], a crystal plasticity model with the information of defects from dislocation dynamics and molecular dynamics simulations was developed and used to study the hardening of neutron-irradiated tungsten. There were no additional fitting parameters needed for the irradiation hardening prediction due to these lower scale simulations.

Multiscale modeling, which plays a key role in linking microscopic structures with macroscopic mechanical properties, is also indispensable for understanding the fundamental mechanisms of material deformation in nanoindentation [165]. Atomistic scale simulations may reveal the mechanisms of dislocation interaction and radiation defects. Mesoscale simulation using dislocation density-based models may shed light on dislocation nucleation, multiplication, and how plastic zones develop and spread. Finally the CPFEM at the macroscale is able to reproduce load–depth curves and other imprint topologies, which can be directly compared with experimental results, to explore the mechanisms of pile-up or pop-in behaviors, and allowing the determination of true material properties by inverse methods.

In Section 4.1, the CPFE models were primarily developed under ambient temperature. As the nanoindentation test is extended to higher temperatures, it becomes challenging to extend CPFE models to higher temperatures as well.

## 5. Summary

Nanoindentation has been widely used in quantifying the mechanical properties of ion-irradiated samples. With the development of experimental and theoretical methods, nanoindentation techniques will continue to promote the understanding of the defect and mechanical behavior evolution of materials used in nuclear energy systems. In this review, practical considerations for nanoindentation testing of ion-irradiated materials, such as the sample mounting for small-sized samples and the test mode to obtain a depth-dependent hardness, were discussed. Issues that may affect the analysis of irradiation hardening were reviewed. These issues, including an ill-defined zero-point, the pile-up effect, and the indentation size effect, arise due to changes in surface morphology and the depth dependence of hardening, as well as inherent complexities in nanoscale indentation. The solutions to these issues were reviewed and suggestions were given. With dedicated consideration, it is expected that nanoindentation will play a key role in the reliable assessment of changes in mechanical properties with ion-irradiation.

The modeling of nanoindentation using the crystal plasticity finite element method for ion-irradiated materials was also reviewed in this paper. In recent decades, the crystal plasticity finite element method has matured, going from a single-crystal model combined with conventional phenomenological theory to a polycrystalline model combined with crystal plasticity theory based on physical mechanisms. Through the physics-based CPFEM, the interaction of dislocations and various defects formed during ion irradiation can be studied for the nanoindentation process. This also helps to unravel the deformation mechanisms and ambiguities behind the indentation size effect in ion-irradiated materials.

Challenges of extending nanoindentation testing to higher temperatures and to multiscale simulation of nanoindentation for ion-irradiated materials were addressed. In view of the progress in high-temperature indentation technique, most of the challenges in high-temperature nanoindentation have been adequately addressed. It is expected that high-temperature hardness measurement will provide a new possibility for analysis of the high-temperature mechanical properties of ion-irradiated materials. It remains a challenge to simulate the irradiation processes in a multiscale manner. It is hoped that in the future, the physics-based CPFEM could be combined with the micro-scale simulation methods, e.g., molecular dynamics, dislocation dynamics, and with models that simulate long-term irradiation defect evolution.

## Figures and Tables

**Table 1:** Challenges and solutions for high-temperature nanoindentation, taken from Ref. [144].

High Temperature Challenges	Solutions
Frame compliance variation with temperature	Watercool frame to prevent changes in frame modulus and/or use high temperature materials with negligible modulus change within temperature range
Sensor/actuator calibrations change with temperature	Remove sensors/actuators from hot zone using insulators and active cooling
Contact surface temperature uncertainty	Measure surface temperature directly with surface mounted thermocouple or thermally instrumented and calibrated indenter, then match indenter temperature to sample surface temperature
Excessive thermal drift during testing	Increase stabilization time or tune indenter/sample temperatures for closer match
Indenter reacts with environment/sample	Exchange indenter material for more chemically stable material, e.g., use sapphire instead of diamond for indenting steels or in oxygen
Sample/indenter oxidizes at temperature	Implement system inside a vacuum chamber or environment chamber with inert gas

## Abbreviations

The following abbreviations are used in this manuscript:

DBTT	ductile to brittle transition temperature
PIE	post irradiation examination
FEM	finite element method
CPFE	crystal plasticity finite element
CPFEM	crystal plasticity finite element method
MSG-CP	mechanism-based strain gradient crystal plasticity
dpa	displacements per atom
BCC	body-centered cubic
FCC	face-centered cubic
RVE	representative volume element
ISO	International Organization for Standardization
ASTM	American Society for Testing and Materials
CSM	continuous stiffness measurement
SC	single-cycle
MC	multi-cycle
PMC	progressive multi-cycle
SEM	scanning electron microscopy
TEM	transmission electron microscopy
AFM	atomic force microscopy
EMC	elastic-modulus-based correction
DGE	damage gradient effect
GND	geometrically necessary dislocation
SSD	statistically stored dislocation
ISE	indentation size effect
RISE	reverse indentation size effect
ATF	accident-tolerant fuel
FMS	ferritic/martensitic steel
RPV	reactor pressure vessel
DBH	dispersed barrier hardening
CRSS	critical resolved shear stress
HR-EBSD	high-angular resolution electron backscatter diffraction
MD	molecular dynamics
KMC	kinetic Monte Carlo
OKMC	object kinetic Monte Carlo
RT	rate theory
DD	dislocation dynamics
